# Family history of esophageal cancer increases the risk of esophageal squamous cell carcinoma

**DOI:** 10.1038/srep16038

**Published:** 2015-11-03

**Authors:** Tiantian Chen, Hongwei Cheng, Xingdong Chen, Ziyu Yuan, Xiaorong Yang, Maoqiang Zhuang, Ming Lu, Li Jin, Weimin Ye

**Affiliations:** 1 Clinical Epidemiology Unit, Qilu Hospital of Shandong University, Jinan, China; 2 Taixing People’s Hospital, Taixing, China; 3 Department of Medical Epidemiology and Biostatistics, Karolinska Institutet, Stockholm, Sweden; 4 Ministry of Education Key Laboratory of Contemporary Anthropology and State Key Laboratory of Genetic Engineering, School of Life Sciences, Fudan University, Shanghai, China; 5 Fudan-Taizhou Institute of Health Sciences, Taizhou, China; 6 Department of Epidemiology, Shandong University, Jinan, China

## Abstract

A population-based case-control was performed to explore familial aggregation of esophageal squamous cell carcinoma (ESCC). Family history of cancer was assessed by a structured questionnaire, and from which 2 cohorts of relatives of cases and controls were reconstructed. Unconditional logistic regression and Cox proportional hazards regression were applied for case-control design and reconstructed cohort design, respectively. We observed a close to doubled risk of ESCC associated with a positive family history of esophageal cancer among first degree relatives (odds ratio [OR] = 1.85, 95% confidence interval [CI]: 1.42–2.41), after adjusting age, sex, family size and other confounders. The excess risks of ESCC increased with the increasing of first-degree relatives affected by esophageal cancer (p < 0.001). In particular, those individuals whose both parents with esophageal cancer had an 8-fold excess risk of ESCC (95% CI: 1.74–36.32). The reconstructed cohort analysis showed that the cumulative risk of esophageal cancer to age 75 was 12.2% in the first-degree relatives of cases and 7.0% in those of controls (hazard ratio = 1.91, 95% CI: 1.54–2.37). Our results suggest family history of esophageal cancer significantly increases the risk for ESCC. Future studies are needed to understand how the shared genetic susceptibility and/or environmental exposures contribute to the observed excess risk.

The data of GLOBOCAN 2012 have shown a great international variation of esophageal cancer incidence, and China is one of areas with the highest esophageal cancer incidence rates in the world^1^. Esophageal cancer has two major histopathological subtypes: esophageal squamous cell carcinoma (ESCC) and esophageal adenocarcinoma. The highest incidence rates of esophageal adenocarcinoma are found in the Western countries, whereas ESCC is the predominant subtype in China, in which more than 90% are of this subtype^2^,^3^. The striking geographic variation implies that environmental factors might be more important in the development of esophageal cancer. However, genetic factors may also contribute to the susceptibility to esophageal cancer, as suggested by familial aggregation^4^,^5^,^6^,^7^,^8^,^9^ and segregation studies^10^,^11^. However, inconsistent results from previous studies have been reported. In addition, according to different study designs, there exist different methods for measuring family history of cancer. In a case-control design, the outcomes are case and control subjects, and the exposure is the disease status in their relatives. In a reconstructed cohort design, the cumulative risks of disease are evaluated for the reconstructed cohorts of relatives of case and control subjects^12^. To date, exploration for the familial risk of esophageal cancer predominantly uses the case-control design, but rarely the reconstructed cohort design.

We thus conducted a population-based case-control study in Taixing of China, a high-incidence area of ESCC, aiming to explore the role of family history of cancer in ESCC occurrence, using two different approaches, i.e. case-control design and reconstructed cohort design.

## Materials and Methods

### Subject recruitment and data collection

We conducted a population-based case-control study on the etiology of esophageal cancer in Taixing of Jiangsu Province from October 2010 to March of 2012. We recruited cases mainly from endoscopy units at the four largest hospitals of Taixing (the People’s Hospital of Taixing, the Second People’s Hospital of Taixing, the Third People’s Hospital of Taixing and the Hospital of Traditional Chinese Medicine of Taixing). More than 90% of the patients in this area are referred to these hospitals and were invited to participate. We also supplemented case recruitment by additional linkage to the local Cancer Registry. During the same time period, we enrolled population controls, which were frequency matched to the cases of ESCC on sex and age (in 5-year groups) and were randomly selected from the Taixing Population Registry. All subjects enrolled in the study were local inhabitants ages 40–85 who have lived in Taixing for at least 5 years prior to diagnosis date for cases or interview date for controls.

From October of 2010 to March of 2012, we found 777 suspected cases from the hospitals, among which 752 were recruited. In addition, by linkage with the local Cancer Registry, 485 cases were further identified among whom 226 died before contact, and 101 refused or were too ill to participate, leaving 158 being recruited into the study. For each case, we also tried to collect sections from formalin-fixed and paraffin-embedded tissue blocks. All sections were stained by H.E. method and reviewed by one study pathologist. Finally we enrolled a total of 775 esophageal cancer cases into the study, and we estimated that about 81% incident cases in the study base were included according to estimated number from local Cancer Registry. Among enrolled cases, 718 (93%) were confirmed histopathologically by the study pathologist, including 648 cases of ESCC, 63 cases of esophageal adenocarcinoma, and 7 cases of other types of esophageal cancer (Supplemental Fig. 1). In total 1311 population-based controls were randomly selected, among whom 221 were excluded due to death before contact, outmigration or unfound, leaving 1090 eligible subjects. Finally, 779 controls were recruited to current study (participation rate: 71.0%).

Trained staff interviewed study subjects face-to-face using a structured questionnaire, which covers information on demographic characteristics, lifestyles and family history of cancer. The staff inquired thoroughly about how many brothers, sisters and children they had, and whether their parents, siblings or children had ever been afflicted with any cancer. For those relatives without a history of cancer, we collected their vital status at the time of interview, including current age or age at death. For those relatives with a history of cancer, we further gathered the information about site of the tumor and age at cancer diagnosis.

### Statistical analysis

We used unconditional logistic regression models to estimate the odds ratios (ORs) with 95% confidence intervals (CIs) of ESCC associated with a family history of cancers of the esophagus, stomach, liver, colorectum, pancreas, and all sites combined. We defined a positive family history of cancer as having at least one first-degree relative affected with cancer. We separately assessed the associations of cancers in parents and siblings with ESCC risk. We also estimated whether cases and controls differed with the number of first-degree relatives with cancer. *P* value for trend was derived from Wald test by entering regression models the number of first-degree relatives with cancer as a continuous variable. In analysis of any cancers, unexposed subjects were those without a positive family history of any cancer. In analysis of digestive cancers, unexposed subjects were those without a positive family history of these cancers. In multivariate analysis, we adjusted for age (continuous), sex, family size (continuous), education (illiteracy/primary school/primary high school/secondary high school and above), tobacco smoking (never/ever smoker of any tobacco), alcohol drinking (never/ever), missing & filled teeth (MFT, none/1 ~ 4/ ≥ 4), times of tooth brushing per day (<2 times/ ≥ 2 times), daily consumption of pickled vegetables (<10 g/ ≥ 10 g) and daily consumption of fresh fruits (<27.5 g/ ≥ 27.5 g).

Based on the kin-cohort data, two cohorts were reconstructed, containing the first-degree relatives of cases and those of controls (171 first-degree relatives were excluded due to missing information of age). Study subjects were followed from birth until the occurrence of esophageal cancer, death, age of 85, or the date of the study interview, whichever occurred first. The exposed cohort included first-degree relatives of cases, and the unexposed cohort those relatives of controls. Cumulative risks of esophageal cancer over time among 2 kin-cohorts were calculated and plotted using Kaplan-Meier method. The relative risk (in term of hazard ratio) of esophageal cancer for the relatives of cases compared to those of controls was derived from Cox proportional hazards regression, which included exposure status (case/control) and sex as covariates, and relative-type (parent/sibling/offspring) as stratification variable. The proportional hazards assumption was checked for each covariate in the Cox model by the method of cumulative sums of Martingale-based residuals^13^, and found not violated. To avoid the influence of familial aggregation, a method proposed by Lee was used to account for the intracluster dependence^14^.

All statistical analyses were carried out with SAS version 9.3 (SAS Institute, Inc., Cary, North Carolina).

### Ethics

This study was approved by the Institutional Review Board of School of Life Sciences, Fudan University and the Institutional Review Board of Qilu Hospital, Shandong University. This study was carried out in accordance with the approved guidelines, and all participants provided written informed consent.

## Results

### Demographics

Current study was based on 648 ESCC cases which were independently reviewed and confirmed by the study pathologist and 779 frequency-matched controls. After deleting 36 records (29 cases and 7 controls) with incomplete questionnaire information on family history of cancer, 619 cases and 772 controls were finally include in the analysis.

Table 1 presents selected characteristics and distribution of potential risk factors among cases and controls. Controls were a bit older than cases, while there was no difference concerning the distribution of sex and education. Compared with controls, cases consumed alcohol more often but brushed teeth less often, while there were no differences concerning smoking, number of missing and filled teeth, and consumption of pickled vegetables and fresh fruits. These factors were considered as potential confounders as well as important risk factors reported in previous literature, and were thus included in the regression model for adjustment in subsequent analyses.

### Family size distribution

Table 2 shows family size, age and sex distributions among siblings and offspring for the case and control groups, respectively. Overall, cases and controls had similar numbers of first-degree relatives (median 8 vs. 8). The median number of siblings and sex distribution were similar between case and control groups, but siblings of control group were on average older than those of case group. For offspring, control group tended to have more offspring than case group, whereas sex distribution and mean age distribution among offspring were similar between the two groups.

### Family history of cancer and risk of ESCC

The first-degree relatives of the cases were more often reported to have been affected by esophageal cancer than those relatives of the controls (34.7% vs. 21.9%), which renders an OR of 1.85(95% CI: 1.42–2.41) (Table 3). The results were consistent when the analyses were limited to parents (OR = 1.63) or siblings (OR = 2.04), separately. The excess risks were evident when the analyses were stratified by type of relatives. The excess risks of ESCC increased monotonically with the increasing number of first-degree relatives reportedly afflicted with esophageal cancer (p for trend< 0.001). In particular, the individuals whose both parents were diagnosed with esophageal cancer had an 8-fold excess risk of ESCC, compared with those without any parents affected by esophageal cancer (adjusted OR = 7.96, 95% CI: 1.74-36.32). However, increasing number of affected siblings did not seem to further increase the relative risks (Table 3). Table 4 shows the associations for family history of cancer, either overall or at selected specific sites, in relation to ESCC risk. Overall 55.6% of cases and 46.6% of controls had a positive family history of any cancer. Excess ESCC risks were associated with a positive family history of any cancer (adjusted OR = 1.43, 95% CI: 1.13–1.81) or digestive tract cancer (adjusted OR = 1.55, 95% CI: 1.23–1.96). Among specific sites, however, although a family history of stomach cancer, pancreas cancer and colorectal cancer tended to increase the risk of ESCC, none of the estimates was statistically significant. The results were similar when family history was examined among parents or siblings separately. However, no association was found when the analysis was limited to offspring, although the number of affected offspring was very small.

### Reconstructed cohort analysis

**Figure 1:**
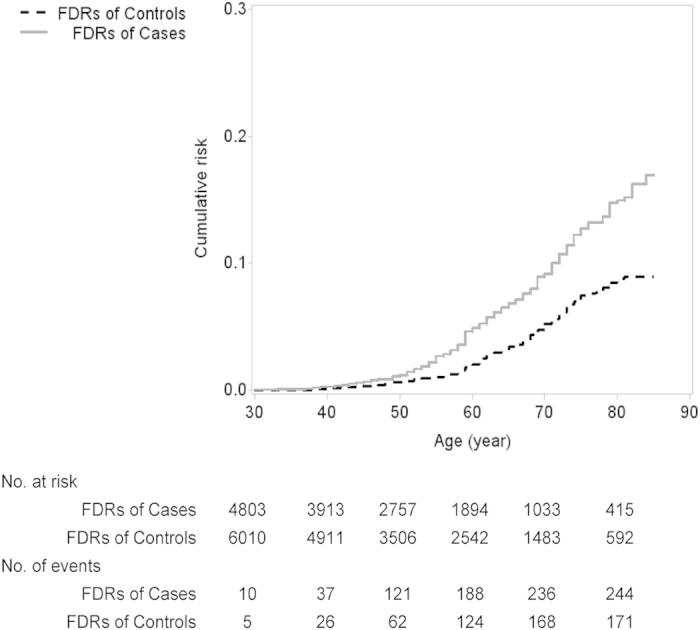
Comparison of cumulative risk of developing esophageal cancer in the first-degree relatives of cases and controls. FDR: first-degree relatives.

Among 4803 first-degree relatives of cases, 244 (5.1%) were reportedly diagnosed with esophageal cancer. The corresponding figure was 2.9% for those relatives of the controls (171 out of 6010). The cumulative risk of esophageal cancer to age 75 was 12.2% in the first-degree relatives of cases and 7.0% in those of controls (hazard ratio = 1.91, 95% CI: 1.54–2.37) (Fig. 1).

### Sensitivity analysis

We also performed a sensitivity analysis by excluding 70 cases whose information of family history was gathered after pathological diagnosis had been made and/or treatment had started (Supplementary Fig 1). In this sub-analysis, 33.91% of cases reported a positive family history of esophageal cancer. The adjusted OR by unconditional logistic regression was 1.79 (95% CI: 1.35–2.37), and the hazard ratio by the Cox regression was 1.90 (95% CI: 1.53–2.37). In another sensitivity analysis, we treated missing family history of cancer as ‘no reported history of cancer’. The results remained virtually unchanged compared with those in the main analysis (data not shown).

## Discussion

In this large population-based case-control study, we confirmed a strong association between a family history of esophageal cancer and the risk of ESCC. The excess risks increased monotonically with increasing number of affected relatives. In particular, if both parents were affected, it rendered an 8-fold excess risk of ESCC for their offspring. By the age of 75, it was estimated that about 12% of the first-degree relatives of ESCC patients might develop the malignancy, while the corresponding figure was 7% for those relatives of the normal control subjects.

Previous epidemiological studies conducted in endemic areas (including mainly Linxian and Shanxi) in China have demonstrated that individuals with a family history of esophageal cancer have a higher risk of such cancer^6^,^8^,^15^. In a case-control study from Turkmen population (another endemic area for ESCC), a more than 2-fold excess risk for esophageal cancer was noted using reconstructed cohort design^4^. However, a multicenter population-based case-control study performed in the United States did not find any familial link for ESCC^16^. A Swedish case-control study also revealed no association between a history of esophageal cancer in first-degree relatives and the risk of ESCC^17^. The inconsistency in results might arise from different frequency of ESCC susceptibility alleles (genetic susceptibility) and variation in attributable environmental or lifestyle risk factors, or a combination of both. It appears that genetic factors play a minor role in the etiology of the “Western type” of ESCC, whereas tobacco and alcohol consumption account for the vast majority of the etiology of this disease^18^. But neither the relative risk associated with smoking nor that of alcohol drinking is of sufficient magnitude to explain the extremely high incidence in China. Because we don’t have exposure information in the first-degree relatives, we cannot distinguish the effects of genetic susceptibility from environmental factors. But for a disease with notable familial aggregation, environmental factors alone cannot account for such a strong aggregation^19^. Recent exome and whole-genome studies also reveal the importance contribution of genetic susceptibility to the occurrence of ESCC^20^,^21^,^22^.

In the present study, we found elevated risks of ESCC among individuals with affected parents (OR = 1.63) or siblings (OR = 2.04) and an obvious dose-response relationship with the increase of affected relatives. The OR was slightly higher for siblings, compared with parents, indicating that recessive or X-linked susceptibility genes might be involved in the occurrence of ESCC, or that siblings share more lifestyle risk factors. In addition, we found the excess risk was remarkably higher if both parents were affected than that of individuals with more than one sibling was affected. This suggested that heredity does seem to take a more important role in the etiology of esophageal cancer in endemic areas.

In many case-control studies of familial aggregation of esophageal cancer, positive family history among first-degree relatives is often used as a risk factor, and odd ratio is calculated. Instead of treating family history information as an “exposure” in case-control design, an alternative analytic strategy is to transform the case-control design into a cohort design. Based on reconstructed cohort design, Akbari *et al.* have estimated the cumulative risk of esophageal cancer to age 75 was 34% in the first-degree relatives of cases and 14% in those of controls in northern Iran^4^. In our study, we observed a similar relative risk, but a much lower absolute risk. Since the incidence rates of ESCC are comparable between the two areas, theoretically we should observe similar cumulative risks among the relatives of control subjects. The more than doubled cumulative risk observed in the previous study might indicate the influence of misclassification of cancer types.

There is controversy about the validity of different designs for assessing familial aggregation. Khoury and Flanders demonstrated that the case-control design would yield a biased risk estimate, with age of relatives and family size being viewed as confounding variables in assessing the disease risk estimate^23^. On the other hand, the reconstructed cohort design is thought to show no such biases. However, Zimmerman concluded that neither family size nor age fulfills the criteria for confounding factors, thus the case-control and reconstructed cohort designs are both valid in estimating familial aggregation of disease^24^. Therefore the investigators should be free to choose the design and measures that best suit the available data to assess familial aggregation. Our results support this notion as similar relative risks were observed for both designs. However, different family history measures may have various implications for counseling using empiric genetic risks. The case-control design is useful when a subject wants to know his/her disease risk given his/her family history. When the disease status of one index person is considered as a risk factor, the reconstructed cohort design is useful to quantify the risk of disease for the relatives of an affected individual.

There are several strengths to this study. First, the detailed information about the family history allowed us to reconstruct cohorts of the relatives of cases and controls, from which we obtained the cumulative risk estimates of esophageal cancer among exposed relatives compared with that among the unexposed relatives. Second, all of the ESCC cases were carefully reviewed and verified by the study pathologist. All subjects underwent detailed in-person interviews which provided necessary information of potential confounders. Finally, the sample size is relatively large and response rates were reasonably high among both cases and controls.

Relying on self-reported information about family history of cancer is a major shortcoming in this study, especially when no validation study has been performed in the study area. This might result in potential misclassification of cancer types in first-degree relatives. However, validation studies in other settings have proved that self-reported family history of cancer could reflect the actual information when compared with retrieving information from medical records^25^,^26^. Study subjects were generally unware of subtypes of esophageal cancer, thus it was impossible for us to distinguish the histological subtypes of esophageal cancers in the relatives. But based on recent report, more than 95% of these esophageal cancers should be ESCC subtype in China^3^. Recall bias is another concern, as case subjects might tend to exaggerate their family history of cancer. However, most cases in this study were enrolled before they were aware of the diagnosis. Further, the similar results from the sensitivity analysis, after we excluded case subjects enrolled after pathological diagnosis being made and/or treatment started, allayed such a concern.

In conclusion, our results indicate that familial aggregation of ESCC in endemic area is notable. The shared genetic susceptibility and environmental exposures, or possibility their interaction, might contribute to this phenomenon which urges future studies to explore the underlying mechanisms.

## Additional Information

**How to cite this article**: Chen, T. *et al.* Family history of esophageal cancer increases the risk of esophageal squamous cell carcinoma. *Sci. Rep.* **5**, 16038; doi: 10.1038/srep16038 (2015).

## Supplementary Material

Supplementary Information

## Figures and Tables

**Table 1:** Demographic information of the study subjects enrolled in a case-control study on esophageal squamous cell carcinoma, Taixing, China.

Variables	Cases (n = 619)	Controls (n = 772)	*P*value*
Age (Mean ± SD, years)	65.9 ± 8.7	67.9 ± 7.9	<0.001
Sex (%)			
Men	431 (70)	548 (71)	0.582
Women	188(30)	224 (29)	
Education (%)			
Illiteracy	203 (33)	238 (31)	0.714
Primary school	242 (39)	296 (38)	
Primary high school	128 (21)	173 (22)	
Secondary high school and above	46 (7)	65 (8)	
Alcohol drinking (%)			<0.001
Never	263 (43)	424 (55)	
Ever	355 (57)	347 (45)	
Missing	1	1	
Smoking (%)			0.538
Never	252 (41)	332(43)	
Ever	366(59)	439 (57)	
Missing	1	1	
MFT†			0.609
None	113 (18)	154 (20)	
1 ~ 4	153 (25)	177 (23)	
≥4	344 (56)	434 (56)	
Missing	9 (1)	7 (1)	
Times of tooth brushing per day			<0.001
<2	489 (79)	512 (66)	
≥2	127 (21)	254 (33)	
Missing	3	6 (1)	
Average daily consumption of pickled vegetables			0.483
<10	243 (39)	292 (38)	
≥10 g	350 (57)	455 (59)	
Missing	26 (4)	25 (3)	
Average daily consumption of fresh fruits			0.698
<27.5 g	290 (47)	371 (48)	
≥27.5 g	280 (45)	374 (48)	
Missing	49 (8)	27 (4)	

^*^P values were based on Wilcoxon rank-sum test for continuous variables, and chi-squared test for categorical variables (two-sided).

^†^MFT referred to sum of missing and filled teeth.

**Table 2:** Distribution of family size, number of siblings and number of offspring in case and control subjects.

	Cases(n = 619)	Controls(n = 772)	*P*value*
Family size, median (range)†	8 (2–16)	8 (2–15)	0.864
Siblings			
No. of siblings, Median (range)	3 (0–12)	3 (0–10)	0.093
No. of brothers (%)	1085(52%)	1298(52%)	0.838 §
Mean age of brothers‡	60.3	61.8	0.008
No. of sisters (%)	1017 (48%)	1202(48%)	
Mean age of sisters ‡	61.5	63.5	0.001
Offspring			
No. of offspring, Median (range)	2 (0–7)	3 (0-8)	0.018
No. of sons (%)	819(53%)	1115(54%)	0.398 §
Mean age of sons‡	41.2	42.0	0.063
No. of daughters (%)	734(47%)	944(46%)	
Mean age of daughters‡	41.4	41.9	0.189

^*^P values were based on Wilcoxon rank-sum test for continuous variables, and chi-squared test for categorical variables (two-sided).

^†^Family size count excluded the index persons.

^‡^Age at the date of interview of their index persons. For those who died before the interview, age at death was used.

^§^P values were based on Chi-squared test comparing the difference between sexes (brothers vs sisters, or sons vs daughters).

**Table 3:** Odds ratios (ORs) and corresponding 95% confidence intervals (CIs) for esophageal squamous cell carcinoma according to family history of esophageal cancer in first-degree relatives*.

Family history of esophageal cancer in relatives	Cases (n = 619)	Controls (n = 772)	OR (95% CI)†	OR (95% CI)‡
First degree relatives				
No	367	545	Reference	Reference
Yes	195	153	1.91 (1.49–2.46)	1.85 (1.42–2.41)
1 affected	150	132	1.70 (1.29–2.22)	1.68 (1.27–2.23)
≥2 affected	45	21	3.28 (1.92–5.62)	2.93 (1.67–5.12)
P for trend			<0.001	<0.001
Parents				
No	439	614	Reference	Reference
Yes	129	102	1.68 (1.26–2.24)	1.63 (1.20–2.20)
1 affected of parents	116	100	1.53 (1.14–2.06)	1.49 (1.09–2.03)
2 affected of parents	13	2	9.11 (2.04–40.72)	7.96 (1.74–36.32)
P for trend			<0.001	<0.001
Father	60	50	1.58 (1.06–2.36)	1.46 (0.97–2.21)
Mother	82	54	2.04 (1.41–2.94)	2.02 (1.39–2.95)
Siblings				
No	483	635	Reference	Reference
Yes	92	64	2.09 (1.48–2.95)	2.04 (1.41–2.94)
1 affected of siblings	82	57	2.07 (1.44–2.97)	2.04 (1.39–2.99)
≥2 affected of siblings	10	7	2.30 (0.86–6.17)	2.04 (0.73–5.69)
P for trend			0.03	0.02

^*^Occurrence of esophageal cancer was very rare in offspring, so the result was not shown.

^†^Adjusted for age (continuous) and sex.

^‡^Adjusted for age (continuous), family size (continuous), sex, education (Illiteracy/primary school/primary high school/secondary high school and above), tobacco smoking (never/ever smoker of any tobacco), alcohol drinking (never/ever), missing & filled teeth (MFT, none/1 ~ 4/ ≥ 4), times of tooth brushing per day (<2 times/ ≥ 2 times), daily consumption of pickled vegetables (<10 g/≥ 10 g) and daily consumption of fresh fruits (<27.5 g/ ≥ 27.5 g).

**Table 4:** Odds ratios (ORs) and corresponding 95% confidence intervals (CIs) for esophageal squamous cell carcinoma according to family history of other types of cancer in first-degree relatives*.

Family history of cancer	First-degree relatives			Parents			Siblings			Offspring			
	Case (N = 619)	Control (n = 772)	OR (95% CI)	Case (N = 619)	Control (n = 772)	OR (95% CI)	Case (N = 602)	Control (n = 730)	OR (95% CI)	Case (N = 590)	Control (n = 732)	OR (95% CI)	
*All cancers combined*													
No	238	358	Reference	340	499	Reference	401	516	Reference	575	701	Reference	
Yes	344	360	1.43(1.13–1.81)	234	223	1.46(1.14–1.87)	181	189	1.32(1.02–1.72)	8	20	0.57(0.24–1.35)	
*Digestive tract cancer*†													
No	273	420	Reference	364	538	Reference	421	553	Reference	576	709	Reference	
Yes	304	293	1.55(1.23–1.96)	206	181	1.57(1.22–2.03)	158	148	1.52(1.15–2.00)	7	12	0.74(0.28–1.97)	
*Stomach*													
No	470	611	Reference	506	667	Reference	532	656	Reference	583	720	Reference	
Yes	82	79	1.36(0.96–1.92)	53	45	1.57(1.01–2.43)	38	40	1.18(0.73–1.92)	0	1	–	
*Liver*													
No	473	601	Reference	524	677	Reference	532	647	Reference	577	713	Reference	
Yes	72	83	1.09(0.76–1.55)	33	33	1.20(0.71–2.03)	36	50	0.89(0.56–1.42)	6	8	0.95(0.31–2.88)	
*Pancreas*													
No	524	673	Reference	550	707	Reference	561	691	Reference	583	721	Reference	
Yes	12	8	2.03(0.79–5.20)	7	4	2.82(0.76–10.54)	5	5	1.20(0.34–4.24)	0	0	–	
*Colorectum*													
No	516	662	Reference	545	702	Reference	557	687	Reference	583	720	Reference	
Yes	21	19	1.33(0.69–2.58)	12	8	1.72(0.68–4.37)	9	10	1.20(0.46–3.11)	0	1	–	

^*^Adjusted for age (continuous), family size (continuous), sex, education (Illiteracy/primary school/primary high school/secondary high school and above), tobacco smoking (never/ever smoker of any tobacco), alcohol drinking (never/ever), missing & filled teeth (MFT, none/1 ~ 4/ ≥ 4), times of tooth brushing per day (<2 times/ ≥ 2 times), daily consumption of pickled vegetables (<10 g/ ≥ 10 g) and daily consumption of fresh fruits (<27.5 g/ ≥ 27.5 g).

^†^Digestive tract cancer includes esophageal cancer, stomach cancer, liver cancer, pancreas cancer and colorectum cancer.
