# Supplementary figures and images for: Family history of esophageal cancer increases the risk of esophageal squamous cell carcinoma
Source: Sci Rep. 2015 Nov 3;5:16038. doi: 10.1038/srep16038 (PMC4630623; doi:10.1038/srep16038)

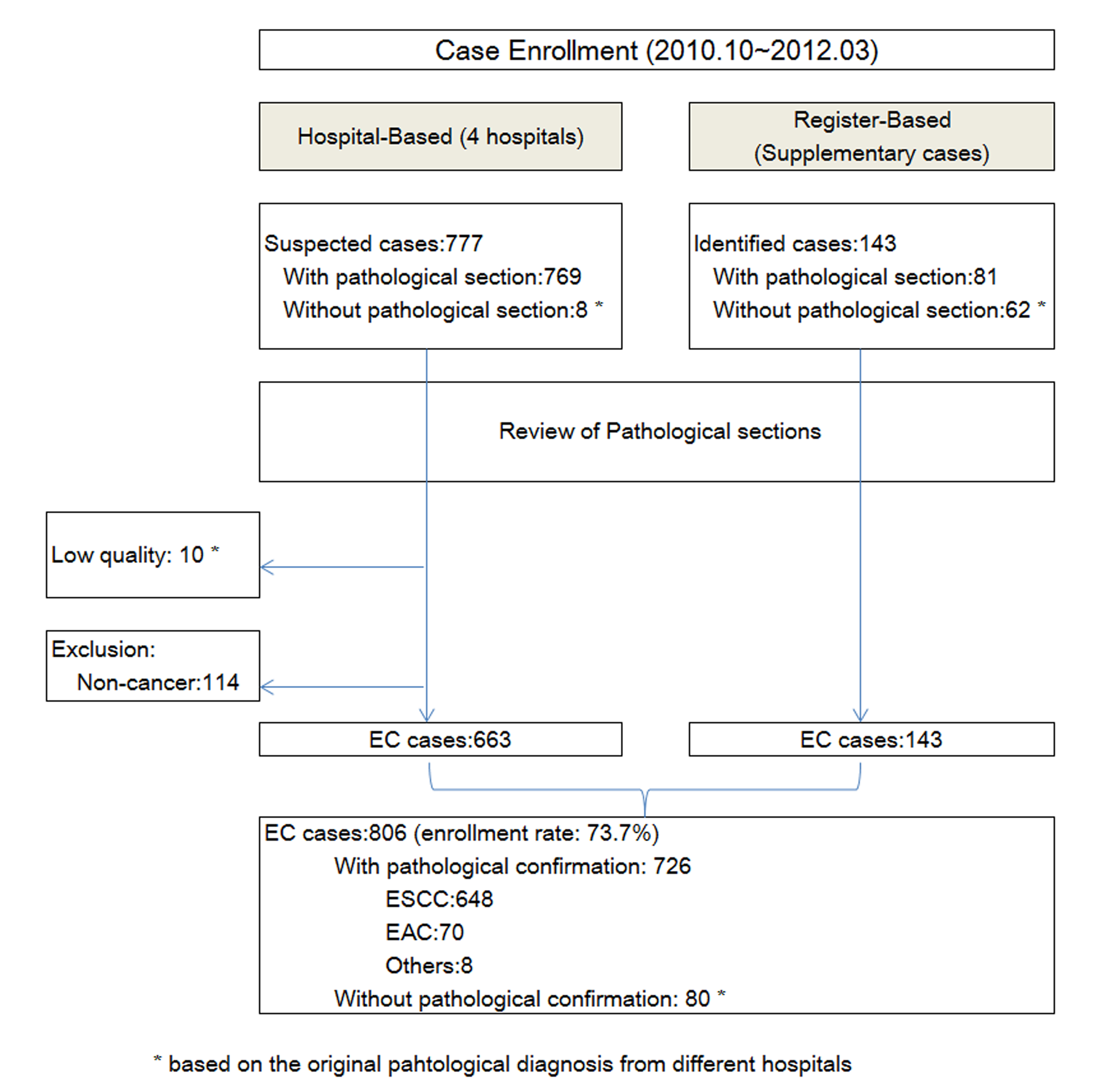

Supplement: Supplementary Information [file srep16038-s1.jpg]
